# Characterization of the Genetic Diversity Present in a Diverse Sesame Landrace Collection Based on Phenotypic Traits and EST-SSR Markers Coupled With an HRM Analysis

**DOI:** 10.3390/plants10040656

**Published:** 2021-03-30

**Authors:** Evangelia Stavridou, Georgios Lagiotis, Parthena Kalaitzidou, Ioannis Grigoriadis, Irini Bosmali, Eleni Tsaliki, Stiliani Tsiotsiou, Apostolos Kalivas, Ioannis Ganopoulos, Panagiotis Madesis

**Affiliations:** 1 Institute of Applied Biosciences, Centre for Research and Technology, Thermi, GR-57001 Thessaloniki, Greece; estavrid@certh.gr (E.S.); glagiotis@certh.gr (G.L.); kalparcha@bio.auth.gr (P.K.); eirinimposmali@certh.gr (I.B.); 2 Institute of Plant Breeding and Genetic Resources, Hellenic Agricultural Organization Demeter, Thermi, GR-57001 Thessaloniki, Greece; y.gregoras@ipgrb.gr (I.G.); tsaliki@ipgrb.gr (E.T.); tsiotsio@pharm.auth.gr (S.T.); kalyvas@ipgrb.gr (A.K.); 3 Laboratory of Molecular Biology of Plants, School of Agricultural Sciences, University of Thessaly, GR-38446 Thessaly, Greece

**Keywords:** *Sesamum indicum*, phenotypic diversity, molecular diversity, high resolution melting, principal component analysis, agglomerative hierarchical clustering, principal coordinate analysis

## Abstract

A selection of sesame (*Sesamum indicum* L.) landraces of different eco-geographical origin and breeding history have been characterized using 28 qualitative morpho-physiological descriptors and seven expressed sequence tag-simple sequence repeat (EST-SSR) markers coupled with a high-resolution melting (HRM) analysis. The most variable qualitative traits that could efficiently discriminate landraces, as revealed by the correlation analyses, were the plant growth type and position of the branches, leaf blade width, stem pubescence, flowering initiation, capsule traits and seed coat texture. The agglomerative hierarchical clustering analysis based on a dissimilarity matrix highlighted three main groups among the sesame landraces. An EST-SSR marker analysis revealed an average polymorphism information content (PIC) value of 0.82, which indicated that the selected markers were highly polymorphic. A principal coordinate analysis and dendrogram reconstruction based on the molecular data classified the sesame genotypes into four major clades. Both the morpho-physiological and molecular analyses showed that landraces from the same geographical origin were not always grouped in the same cluster, forming heterotic groups; however, clustering patterns were observed for the Greek landraces. The selective breeding of such traits could be employed to unlock the bottleneck of local phenotypic diversity and create new cultivars with desirable traits.

## 1. Introduction

Sesame (*Sesamum indicum* L.) is an ancient oilseed crop of the Pedaliaceae family and its seeds are considered a commodity of great commercial importance. In the last decade sesame has been classified second after tea and above coffee as well as ninth among the major oil crops as per the annual import quantity worldwide [1]. Sesame has long been considered as a ‘super food’ and is used in traditional food products such as pastels and tahini paste owing to its high caloric value and richness in nutrients as well as its nutraceutical and pharmaceutical properties [2]. The high nutritional value of sesame seeds derives from the high content of polyunsaturated fatty acids, antioxidants (sesamolin, sesamin and sesamol) and proteins [3,4]. 

Sesame is cultivated in more than 11.28 million hectares per world crop harvested area with an annual production of approximately 5.86 million tons from 2015–2019 [1]. It is mainly cultivated in the tropical and semi-tropical regions of Asia, Africa and South America. In Greece, sesame cultivation declined after 1980; however, the extensive and cumulative knowledge on the nutritional and health benefits of sesame seeds along with the market demand for sesame seeds and oil have led to an increasing interest in both cultivation and production. Considering the ability of sesame to adapt to different environments especially in semi-arid regions [5] and the low irrigation requirements, it could be an ideal alternative for crops that cannot be productive in marginal lands and especially in the current state of climate change and the negative effects on agriculture [6,7]. Nevertheless, the growth and productivity of the sesame crop are greatly affected under adverse environmental conditions and efforts have been made to identify major genes for a targeted improvement of the crop towards enhancing tolerance to multiple abiotic stresses [8]. Phenotypic and genetic diversity are key factors for the effective protection and improvement of traditional varieties [9] such as sesame landraces. Previous studies have shown that sesame landraces have a large range of diversity in both agro-morphological and genotypic characteristics [6,10,11,12,13].

To identify agro-morpho-physiological traits that contribute to the total diversity and characterize the levels of similarity among cultivars, an investigation of the phenotypic diversity and structure is essential [14,15,16,17]. Morphological traits such as growth habit, plant height, primary and secondary branches, leaf arrangement, leaf shape, hairiness of the stem flower and capsule, number of flowers per leaf axil, number of capsules, number of carpels per capsule, days to maturity, seed yield and seed coat color [10,18,19] were considered as the most representative agro-morphological traits used to estimate the genetic diversity in sesame. However, a diversity analysis solely based on the direct and cost-effective approach of morphological evaluation is biased due to being subjected to environmental influences and the overall complexity of various morphological traits [18,20].

The genetic diversity in sesame has been thus far detected by universal markers such as random amplified polymorphic DNA (RAPD) [21,22,23,24,25], amplified fragment length polymorphisms (AFLPs) [26,27,28], inter simple sequence repeats (ISSRs) [29,30], genome sequence-simple sequence repeats (gSSRs) [12,31,32,33,34], chloroplast SSR (cpSSRs) [35] and expressed sequence tag-SSRs (EST-SSRs) [35,36,37,38,39,40,41]. SSRs are considered an ideal marker choice for assessing the genetic diversity and germplasm characterization considering their high-fidelity, co-dominant nature, chromosome specificity, high polymorphism and reproducibility [42,43]. SSR markers were previously used for assessing the genetic diversity in sesame demonstrating an extensive genetic divergence [44].

Different cultivars can be used to preserve the gene pool and design an effective reproductive strategy towards sesame crops with a high seed quality and increased adaptation to the Greek climatic conditions. Nevertheless, there is no available information on the genetic diversity of the Greek sesame cultivars. The main aim of this study was to characterize the available genetic diversity of the Greek sesame landraces in comparison with other genetic material from the Mediterranean basin and Asia by phenotypic and molecular descriptors. More specifically, the phenotypic characterization was performed using 28 morpho-physiological parameters whereas the molecular characterization involved seven EST-SSR markers coupled with a high-resolution melting (HRM) analysis, which can detect even single nucleotide polymorphisms (SNPs), using a whole amplicon sequence for inferring variations. An integrative assessment of phenotypic and genetic variation is a key component for crop improvement and of paramount importance for effective conservation and the development of breeding strategies [45,46].

## 2. Results

### 2.1. Morpho-Physiological and Agronomical Diversity of the Sesame Landraces

**Figure 1:**
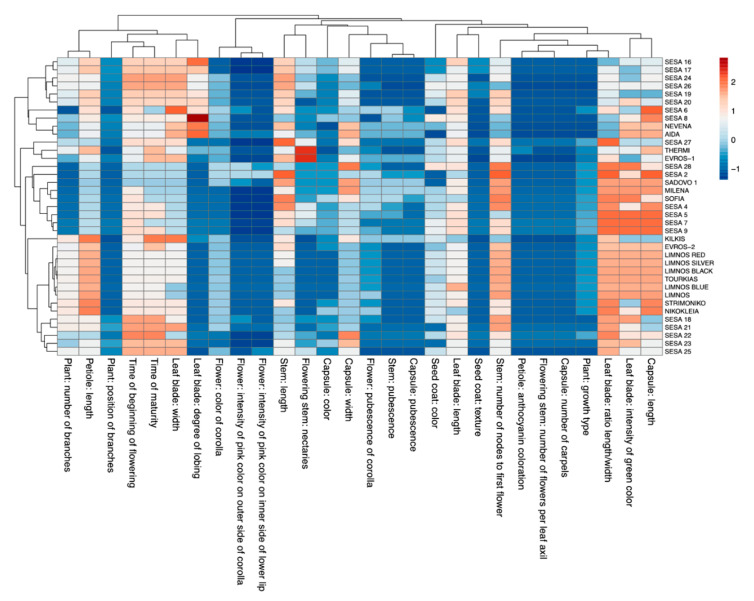
Heatmap of the 28 morpho-physiological descriptors for each of the 37 sesame cultivars. A hierarchical clustering heatmap was performed on log-transformed qualitative data. The color-coded scale indicates an increase (red) and a decrease (blue).

To assess the phenotypic diversity of the 37 sesame cultivars, we used 28 qualitative morphological and agronomical characteristics related to growth, branching, leaf and stem morphology, flower, capsule and seed traits as well as flowering time and seed maturity based on the guidelines of the International Union for the Protection of New Varieties of Plants for *Sesamum indicum* L. [47] (https://www.upov.int/edocs/tgdocs/en/tg292.pdf (accessed on 15 September 2018)). The raw data of the qualitative characteristics are presented in Table S1 Climatic data were also monitored for the cultivation area and are shown in Table 1. The sesame cultivars showed extensive diversity in the morpho-physiological traits with the most prominent ones being: (i) time of maturity, (ii) time of beginning of flowering, (iii) leaf-related traits (leaf blade width, leaf blade ratio length/width, degree of lobing, petiole length and intensity of green color), (iv) stem-related traits (stem length and number of nodes to first flower) and (v) capsule width and length (Figure 1). 

Several cultivars exhibited a large variation for specific traits. More specifically, a greater degree of leaf blade lobing was observed for the cultivar “SESA 8” from Nepal, “AIDA” and “NEVENA” from Bulgaria and “SESA 16” from Yemen compared with the rest of the cultivars. Furthermore, “THERMI” and “EVROS-1” showed the highest values in flowering stem nectaries. A pattern was also observed among the 37 cultivars for the leaf blade ratio length/width and the intensity of green color as well as the capsule length. Most of these cultivars showed increased values for these traits except a few of the Yemen cultivars, “EVROS-1” and “KILKIS”. Notably, the Greek cultivars were overall clustered for the majority of the traits except “KILKIS”, which showed a distinct pattern in the morpho-physiological variables (Figure 1).

A Spearman correlation matrix revealed a wide range of positive and negative correlations among the studied traits except Stem: number of nodes to first flower, Stem: length and Petiole: anthocyanin coloration, which did not show any significant correlation (Table S2). High significant positive correlations were observed between unrelated traits such as capsule pubescence and stem pubescence (0.924) as well as seemingly related traits such as those pertaining to the flower color (corolla and inner lip) and the beginning of the flowering with time of maturity (Table S2). The most significant negative correlations were observed between the leaf blade ratio (length/width) and the degree of lobing (−0.995) as well as the plant growth type with the position of branches (−0.943). The plant growth type was also negatively correlated with leaf blade width (−0.701), time of beginning of flowering (−0.730) and degree of lobing (−0.616). Other significant correlations included: (i) the number of branches positively correlated to the petiole length (0.867) and the time of maturity (0.663); (ii) the position of branches positively correlated with leaf blade width (0.754), time of maturity (0.613), degree of lobing (0.646) and beginning of flowering (0.695) and negatively correlated with the leaf blade ratio length/width (−0.625); (iii) leaf blade width was positively correlated with the degree of lobing (0.675) and time of maturity (0.642), but it was negatively correlated with the leaf blade ratio length/width (−0.671); (iv) flower pubescence of the corolla was strongly correlated with capsule pubescence (0.620) and the beginning of flowering (−0.657) (Table S2).

**Figure 2:**
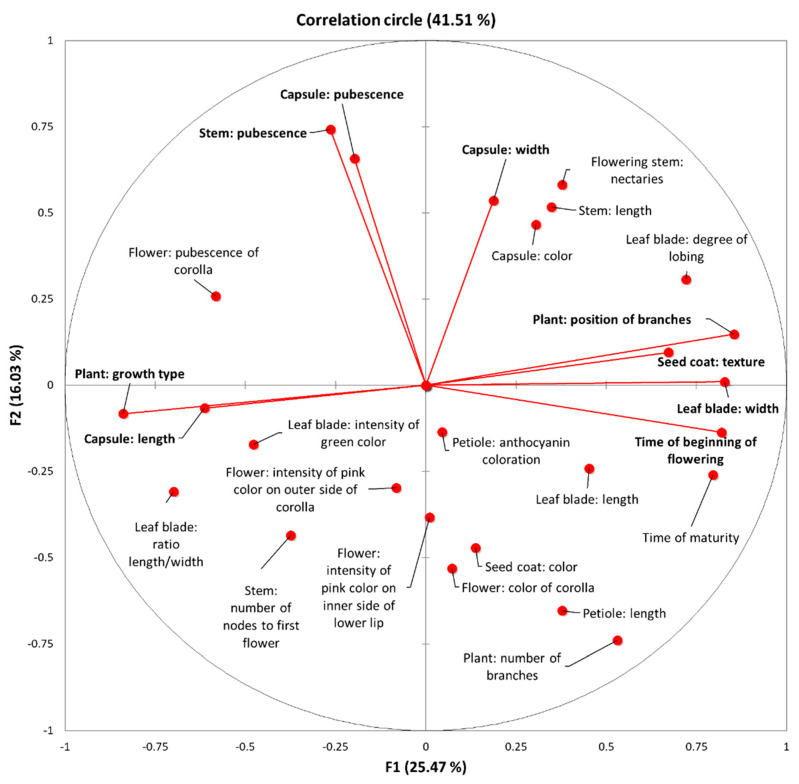
Correlation circle depicting the initial variables for the 37 sesame cultivars. Dots represent the projection of the different variables in the F1 and F2 components’ space. Variables with high squared cosine values, linked to the corresponding axes, are represented with red lines.

A principal component analysis (PCA) was implemented to identify the most important morpho-physiological and agronomical traits for assessing the sesame cultivar variation. We obtained eight significant factors with an Eigenvalue > 1 using Kaiser’s criterion [48], which explained 78.85% of the total variation (Table 2). The first two components represented 41.51% of the initial variability of the morpho-physiological data. The correlation circle depicted the projection of the initial variables in the space of F1 and F2 components (Figure 2). Based on the correlation matrix and the correlation circle on axes F1 and F2, the first component, which accounted for 25.47% of the total variation, included the growth type, position of branches, leaf blade width, time of beginning of flowering, capsule length and seed coat texture. The second component, which explained 16.03% of the total variation, was mainly correlated to traits such as stem pubescence, capsule pubescence and capsule width.

**Figure 3:**
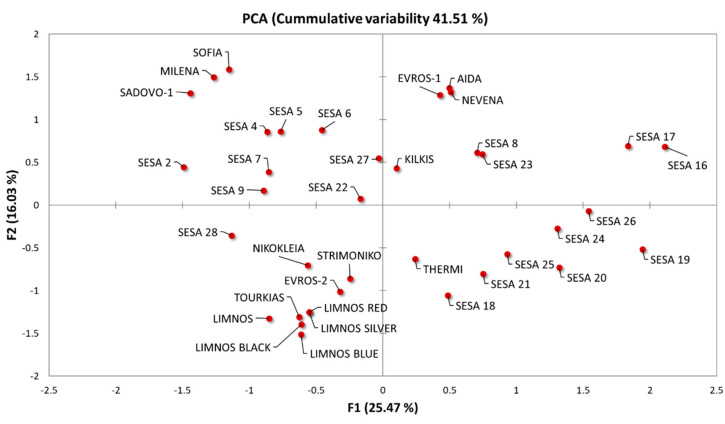
Principal component analysis (PCA) biplot based on the morpho-physiological parameters of the 37 sesame cultivars regarding the first two principal components (F1 and F2).

The distribution of the samples along the first two axes on the PCA biplot revealed the phenotypic variation among the 37 cultivars (Figure 3). The plot grouped the cultivars according to their phenotypic similarity based on the analyzed characteristics. Landraces from different origins were generally scattered covering almost the whole variation spectrum along F1 and F2. However, we observed a broad clustering in the majority of the Greek sesame cultivars except “EVROS-1”, which formed a unique cluster with the Bulgarian cultivars “AIDA” and “NEVENA”. Additionally, the morpho-physiological characteristics of the “SADOVO-1”, “MILENA” and “SOFIA” cultivars were probably unique. Most of the sesame cultivars from Yemen also formed a separate group (Figure 3).

**Figure 4:**
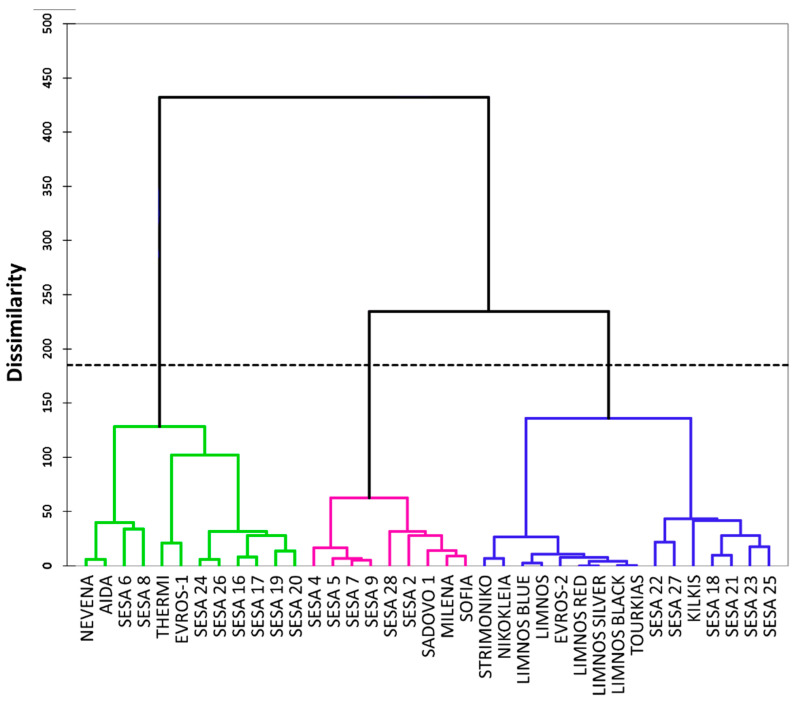
Dendrogram of the agglomerative hierarchical clustering (AHC) for the 37 sesame cultivars based on 28 morpho-physiological traits.

Unsupervised agglomerative hierarchical clustering (AHC) based on Ward’s method was used to group the available data into clusters of increasing dissimilarity. The dendrogram of Figure 4 shows the 37 cultivars split into three groups. Group 1 (displayed in the green color) contained 12 cultivars whereas group 2 (displayed in the magenta color) had nine cultivars and group 3 (displayed in the blue color) included 16 cultivars. Among the 37 cultivars from different regions of origin, there were no specific clusters based on the source of origin or locality except for the majority of the Greek cultivars, which were clustered into group 3 (displayed in the blue color) (Figure 4). The highest distance between the class centroids was observed in groups 1 and 2 (9.24) followed by groups 2 and 3 (7.07) and 1 and 3 (6.48). The sub-cluster of group 3 (displayed in the blue color) containing the Greek cultivars was more homogeneous (flatter in the dendrogram) compared with the other sub-cluster of the same group and the other two groups, which was also confirmed by the relatively low within-class variance (23.8). Similarly, group 1 also showed the lowest within-class variance (21.8) compared with group 2 (38).

### 2.2. Genetic Diversity of the Sesame Landraces Based on the EST-SSR Marker Analysis

To further investigate the genetic diversity among the sesame landraces, we used seven EST-SSR markers for the molecular characterization of the 35 sesame landraces excluding “AIDA” and “NEVENA” due to a limited seedstock and low germination efficiencies. Different HRM profiles of the seven EST-SSR markers were used for scoring the derived binary data. To test whether the selected seven microsatellite loci were informative to distinguish the sesame landraces, a statistical re-sampling showed that these microsatellite loci were sufficient to ensure identification. According to the discriminating power value for each locus, we tested combinations starting with the most discriminating and adding one locus at each step. The optimal combination of seven EST-SSRs allowed for the discrimination of all of the analyzed genotypes. Using this locus combination, we observed a low probability of identity (PI = 3 × 10^−5^). Overall, the markers showed 98.39% of polymorphic loci with a mean diversity (h) for all loci of 0.172 ± 0.015 and an unbiased diversity (uh) of 0.177 ± 0.016. Table 3 shows the diversity statistics for the seven EST-SSR markers used to assess the genetic diversity of the 35 sesame landraces. The polymorphism information content (PIC) values ranged from 0.65 for the ZM_10 marker to 0.89 for marker ZM_47 with an average PIC value of 0.82, which indicated that the selected EST-SSR were highly polymorphic. Regarding heterozygosity (h), the highest (0.21 ± 0.06) and lowest (0.148 ± 0.032) values were observed for ZM_22 and ZM_47, respectively. The maximum Shannon’s Index (I) value (0.345 ± 0.072) was observed for marker ZM_22 whereas the lowest was in ZM_47 (0.268 ± 0.045).

**Figure 5:**
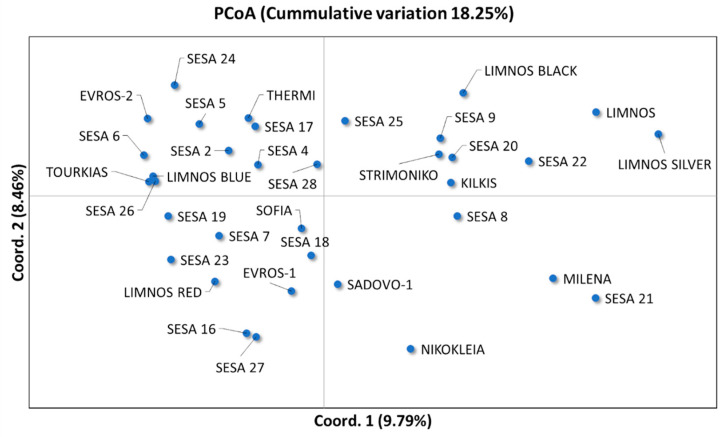
Principal coordinate analysis (PCoA) of the first two coordinates (Coord. 1 and Coord. 2) based on the EST-SSR data for the 35 sesame landraces.

**Figure 6:**
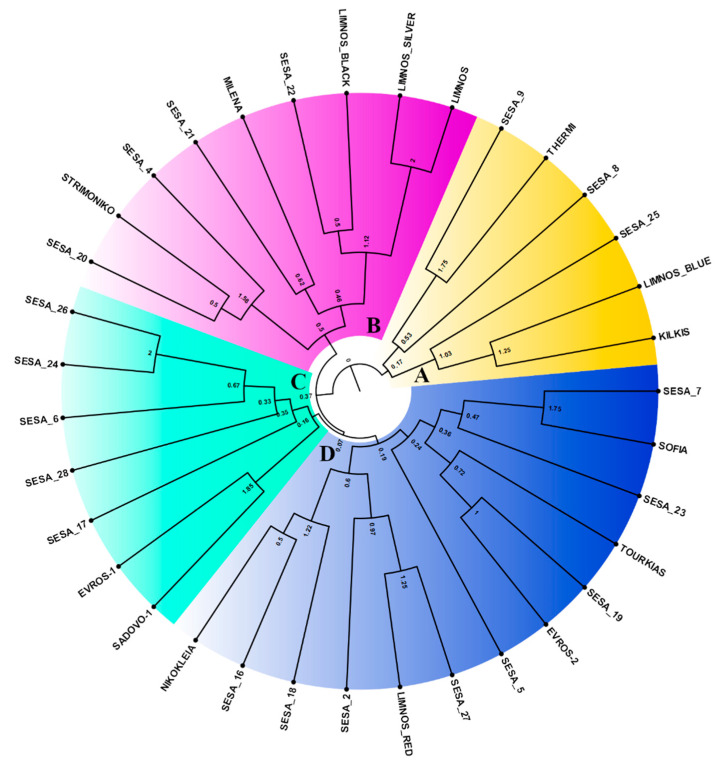
Unweighted Pair Group Method with Arithmetic mean (UPGMA) dendrogram of the 35 sesame landraces based on the genetic distance matrix derived from the EST-SSR data. The formed clades of the sesame landraces were color-coded (clade A: yellow, clade B: magenta, clade C: cyan, clade D: blue).

Unique genotypes on a two-dimensional multivariate space were predicted generating at least two major clusters, which were separated by the central axis of coordinate 1 (Figure 5). The first two coordinates of the principal coordinate analysis (PCoA) explained 18.25% of the total variation among the 35 sesame landraces (Figure 5) whilst the first three coordinates covered up to 26.14% of the cumulative variance. However, we did not observe any significant association between the landraces based on their region of origin. The sesame landraces in the Unweighted Pair Group Method with Arithmetic mean (UPGMA) clusters were grouped into at least four major clades, A–D (Figure 6). The UPGMA method revealed the early divergence of clade A (Figure 6), which included landraces that were distinctively clustered in the upper right quartile of the PCoA (Figure 5). Similarly, the landraces of clade B showed clear clustering in both analyses (Figure 6). Nevertheless, the landraces of the closely related C and D sister clades overlapped in the PCoA (Figure 5). Notably, the majority of the Greek landraces such as “LIMNOS”, “LIMNOS BLUE”, “LIMNOS SILVER”, “LIMNOS BLACK”, “KILKIS”, “THERMI” and “STRIMONIKO” were present in the early diverging clades A and B and were grouped in the upper half of the PCoA in contrast to the discrepancies observed in the “EVROS-1”, “EVROS-2” and “LIMNOS RED” landraces between the PCoA and the UPGMA tree (clades C and D).

## 3. Discussion

An accurate knowledge of phenotypic and genetic diversity is key to the effective use and preservation of traditional varieties, which are at a high risk of extinction. Domestication, plant breeding and genetic drift have likely limited the genetic basis of cultivated sesame leading to a reduced genetic variation [40]. Thus, the preservation of the sesame germplasm is of paramount importance for introducing a new variation into the available gene pool and for the development of breeding strategies towards more resilient sesame crops. Herein, we have identified the key components that contribute the most to the diversity of the, thus far, uncharacterized Greek sesame landraces based on qualitative morpho-physiological and genetic approaches. Additionally, landraces from different eco-geographical regions were used to investigate potential genetic similarities with the local cultivated landraces.

The morpho-physiological parameters used herein were rather effective in discriminating the studied sesame cultivars and revealing the underlying phenotypic diversity. The major determinants of the genetic diversity in the studied landraces were growth type, position of branches, leaf blade width, time of beginning of flowering, capsule length and seed coat texture, stem pubescence, capsule pubescence and capsule width (Figure 1 and Table S2). Flowering initiation was also a major determinant of genetic diversity according to Furat and Uzun [10], along with days to emergence, capsule initiation and seed yield. Additionally, seed coat color ranging from white to black was reported to be a highly polymorphic trait in sesame cultivars [49,50] yet in our study, seed coat color was less variable among the landraces (Figure 1). A high correlation coefficient was also observed for the flower pigmentation traits and leaf profile, as reported by Prasad and Gangopadhyay [50] and Pandey et al. [49]. Based on our results, so were leaf shape-related traits (length/width ratio, degree of lobing, intensity of green color) whereas the flower pigmentation-related traits were not. Flowering stem nectaries were also highly variable with the Greek landraces “EVROS-1” and “THERMI” having the highest values (Figure 1), which is an important trait for pollination and therefore could be essential in breeding strategies for an improved yield [51].

Significant correlations were revealed in a wide range of the studied traits such as the positive correlations between capsule and stem pubescence as well as flower pigmentation and flowering initiation with time of maturity. Interestingly, traits related to pubescence of the different reproductive and vegetative sesame organs/tissues showed high correlation coefficients [50]. However, the negative correlation observed between flowering initiation with the less variable traits of vegetative and reproductive pubescence could be the effect of an early artificial selection during the breeding of sesame cultivars. One of the most important agronomic traits in sesame breeding includes the time of maturity, given it is associated with plant yield. Our analysis showed a strong positive correlation of sesame maturation time with branching traits and leaf blade width, which could be effectively used in breeding for both early and late maturity in sesame cultivars.

Based on the correlation analysis of the 28 qualitative morpho-physiological traits, we observed that landraces of the same geographical origin were not always clustered together, as was also previously reported by Tabatabaei et al. [52] and Pandey et al. [49]. However, several landraces of the same geographical origin such as most of the Greek landraces seemed to form distinct sub-clusters (Figure 3 and Figure 4), suggesting that geographical origin may potentially influence cluster composition. Nevertheless, sub-clusters such as those of “THERMI” and “EVROS-1” were clustered independently from other landraces of the same origin, which may be explained by differences in their parental morpho-physiological traits, in outcrossing rates and in selection strategies [21].

Regarding the assessment of the molecular genetic diversity among the 35 sesame cultivars, the selection of the seven EST-SSR markers was based on the work by Wei et al. [38] showing a significant amount of polymorphisms among 24 sesame accessions. The aim was to have the largest possible coverage of different types of SSR polymorphic motives with the highest possible PIC value as observed by Wei et al. [38]. Herein, the selected seven EST-SSR markers coupled with the HRM analysis were also proven to be highly informative, robust and polymorphic as supported by the PIC and Shannon’s index values (Table 3). Similar results of highly polymorphic SSR markers with average PIC values > 0.5 have been previously reported [32,53] in contrast with the low average PIC values in other genetic analyses of sesame germplasm (0.42–0.52) [34,40,54]. Similarly, to Ramprasad et al. [18], we also observed a low average heterozygosity of 0.172 in the 35 sesame cultivars, which was possibly attributed to the self-pollinated nature of the crop.

Although the association between genetic similarity and geographical proximity has been previously reported in sesame [7,55,56]; herein, a high degree of genetic variation among the studied cultivars did not corroborate with geographical distribution (Figure 5 and Figure 6). The absence of any evident clustering among the sesame cultivars originating from the same region was also observed by Wu et al. [40] and Bhattacharjee et al. [19]. Nevertheless, a broader clustering was observed for specific sesame cultivars such as “LIMNOS”, “LIMNOS BLACK”, “LIMNOS SILVER”, “STRIMONIKO”, “KILKIS” and “SESA 8, 9, 20, 22” (Figure 5). Another group was the “MILENA” and “SESA 21” cultivars (Figure 5). Notably, the “NIKOKLEIA” landrace from Cyprus did not cluster with other cultivars (Figure 5), which could be attributed to the potentially low gene flow as a result of the geographic isolation of the island.

This uncoupling of geographic and genetic diversity was also revealed by the UPGMA dendrogram (Figure 6). For instance, the five cultivars from Limnos in the north Aegean (Greece) were split among three of the major tree clades, indicating that despite their common origin they were rather genetically diverse. Similar patterns were prominent for the “EVROS-1” and “EVROS-2” sesame cultivars in contrast to those from central Macedonia (Greece) (“THERMI”, “KILKIS”), which were grouped in the same clade A yet distinctively variable from “STRIMONIKO”. Interestingly, the Yemen cultivars showed an even broader distribution in all four clades. Similarly, sesame accessions from different geographical origins were also scattered throughout the UPGMA clusters and the two-dimensional PCA space [27], which was also observed by Woldesenbet et al. [57], suggesting a lack of association between geographical origin and population differentiation. This lack of association between geographical origin and genetic diversity observed across different sesame landraces could be explained by the distribution of sesame seeds via markets and migration routes across widely separated locations [58].

In terms of diversity, the phenotypic and molecular analyses of the 37 sesame cultivars based on 28 qualitative traits and seven EST-SSR markers revealed inconsistencies between the genetic variation and geographical origin, which was in accordance with similar studies when assessing the genetic diversity of sesame collections from China [11] and India [18]. Estimates of genetic variation based solely on DNA marker analyses failed to infer evolutionary potential and the ability of plants to cope with environmental change. Therefore, the molecular evaluation of genetic diversity may not fully explain the quantitative genetic variability [11]. As such, the assessment of the phenotypic variation is also essential for revealing functional diversity influenced by environmental factors. Based on the above, the assessment of genetic variation necessitates a more integrative approach using both phenotypic and molecular descriptors especially when selecting diverse landraces from heterotic groups for breeding programs.

## 4. Materials and Methods

### 4.1. Plant Material

The plant material used in this work included a total of 37 sesame (*Sesamum indicum* L.) landraces from Greece, Bulgaria, Italy and several Asian countries (Table 4). The 11 Greek landraces used herein were traditional Greek cultivars and represented the total diversity of the Greek sesame cultivars. The plant material was acquired from German, Bulgarian and Greek gene banks. For the morpho-physiological characterization of the studied cultivars, plants were grown in the Hellenic Agricultural Organization-DEMETER, Institute of Plant Breeding and Genetic Resources (Thermi, Thessaloniki, Greece). The genetic analysis of the different landraces was performed at the Institute of Applied Biosciences (INAB)/Centre for Research and Technology Hellas (CERTH).

### 4.2. Morpho-Physiological Analysis of Sesame Landraces

For the morpho-physiological analysis, field trials were carried out for two consecutive seasons (2016–2017) at the experimental station of the Hellenic Agricultural Organization-DEMETER, Institute of Plant Breeding and Genetic Resources (Thermi, Thessaloniki, Greece), latitude 40°32′49.63′′ N, longitude 23°01′10.81′′ E. Plants were grown in a silty loam soil with 24% silt, 22% clay, 54% sand and 2.36% organic matter with a pH of 7.9. The accessions were grown in 5 m long two-row plots with a row to row spacing of 70 cm and plant to plant spacing of 10 cm. Ten plants were randomly selected and tagged in each plot to determine the growth parameters (*n* = 10). A total of 28 morphological and agronomical characteristics related to growth, branching, leaf and stem morphology, flower, capsule and seed traits as well as flowering time and seed maturity (Table S2) were recorded for each plot based on the guidelines of the International Union for the Protection of New Varieties of Plants [47] (https://www.upov.int/edocs/tgdocs/en/tg292.pdf, accessed on 15 September 2018). The raw data of the qualitative characteristics are presented in Table S1. For each landrace, the respective states of expression of each characteristic were demonstrated (Table S1). Additionally, climatic data (mean maximum temperature, minimum temperature and total rainfall) of the institute’s location were monitored during the sesame cultivation period from May to September for the years 2016–2017.

Morphophysiological data were analyzed using XLSTAT software (version 2014.1; Addinsoft Inc., New York, NY, USA) and the webtool ClustVis [59] for the hierarchical clustering heatmap. For the classification of landraces based on the qualitative traits, a PCA was applied after a standardization of the variables and the Spearman coefficient was used to assess the correlation among the variables. The covariance matrix was then used to determine the principal components of the data. Within the PCA, factor loadings higher than 0.55 were regarded as significant and biplots were constructed with regard to the first two most important principal components. Additionally, Ward’s method was used for the agglomerative hierarchical clustering (AHC).

### 4.3. DNA Isolation, PCR Amplification and HRM Analysis

Total DNA was isolated from 100 mg of dried leaf tissue using the modified CTAB protocol described by Doyle and Doyle [60] The DNA samples were re-diluted in a 1X TE buffer (10 mM Tris-Cl pH 8.0, 1 mM EDTA) at a final working concentration of 20 ng μL^−1^. DNA quantity and quality were assessed by gel electrophoresis in 1% agarose gel and spectrophotometrically using a UV-Vis Spectrophotometer Q5000 (Quawell Technology Inc., San Jose, CA, USA).

The genetic characterization was performed for 35 out of the total 37 sesame cultivars shown in Table 1. Landraces NEVENA and AIDA were excluded from the molecular analysis due to the limited available seedstock with a low germination efficiency. The analysis was performed using seven EST-SSR (ZM_2, ZM_10, ZM_11, ZM_21, ZM_22, ZM_34 and ZM_47) markers with high transferability previously described by Wei et al. [38], which were selected to cover different polymorphic SSR motifs with the highest possible PIC value. PCR amplification, DNA melting, HRM and end-point fluorescence-level determination along with an EST-SSR analysis were performed in a Rotor-Gene 6000 real-time 5-Plex HRM PCR thermocycler (Corbett Research Pty Ltd., Sydney, Australia) using the Rotor-Gene Q software (version 2.0.2) (Qiagen, Germantown, MD, USA). PCR reaction mixtures were prepared in a final volume of 20 µL containing 1 × KAPA Taq Buffer (Kapa Biosystems, Wilmington, MA, USA), 1.5 mM MgCl_2_, 0.2 mM dNTPs, 0.6 mM of each primer, 1.5 mM Syto^®^ 9 green fluorescent nucleic acid stain, 1 U Kapa Taq DNA polymerase and 40 ng of the DNA template. Cycling was carried out with an initial denaturation step at 94 °C for 4 min followed by 40 cycles at 94 °C for 40 s, 55 °C for 40 s and 72 °C for 1 min. The HRM was performed by an initial pre-melt conditioning of the PCR products at the first appropriate temperature for 90 s followed by a melting ramp from 70 to 95 °C, with 0.1 °C increments every 1 s. The normalized raw and negative derivative of fluorescence (F) over temperature (T) (dF/dt) melting curves were used for sample comparisons. For all of the primer pairs, detection sensitivity and reproducibility tests were confirmed by the replicated DNA samples. The differences between the studied sesame landraces were investigated via the shape of the melting curve profiles. Samples were grouped based on their similarity with representative standard curves among the samples for each EST-SSR marker.

### 4.4. EST-SSR Genotyping and Data Analysis

The genetic diversity of the sesame landraces was assessed for 35 out of the total 37 sesame cultivars excluding “AIDA” and “NEVENA”. The HRM profiles of each sample were compared against the representative standard curves of each marker. Scoring was performed in a binary fission with “1” indicating a HRM curve similarity with the standard curve > 70% and “0” indicating the absence of similarity. The GenAlex 6.5 software package [61] was used to generate a pairwise genetic distance matrix based on the binary scoring data. Shannon’s information index (I), the expected Heterozygosity (He), unbiased diversity (uh) and the polymorphism information content (PIC) were subsequently calculated. The resulting matrix was further used to perform a principal coordinate analysis (PCoA) using the GenAlex 6.5 software [61] and dendrogram reconstructions using the MEGA software version 4.0 [62] and FigTree version v1.4.4 (http://tree.bio.ed.ac.uk/software/figtree/ (accessed on December 2019)).

## 5. Conclusions

The characterization of the genetic diversity present in the Greek sesame landraces is essential for assisting further research in breeding approaches and the selection of parental lines. By analyzing a broad spectrum of qualitative morpho-physiological traits and EST-SSR markers coupled with an HRM, we identified phenotypic and molecular genetic diversity for a collection of sesame landraces originating from the Mediterranean basin, especially Greece, and Asia. Overall, our analysis revealed that not all genotypes from the same geographical origin were grouped in the same cluster. Therefore, the landraces from such heterotic groups could be used for enriching diversity in crossbreeding programs for favorable sesame cultivation with an increased adaptation to a range of climatic conditions. Plant growth type, position of branches, leaf blade width, stem pubescence, time of beginning of flowering, capsule traits and seed coat texture were identified in this study as being highly variable in describing the morpho-physiological diversity of the studied landraces. The selective breeding of such traits could be employed for the development of sesame populations to unlock the bottleneck of local phenotypic diversity and create new cultivars with desirable traits. Additionally, these data could be effectively implemented in breeding programs for the preservation of the genetic material of the Greek landraces and also in granting community plant variety rights (CPVR) for registered new sesame varieties in the EU Community Plant Variety Office (CPVO).

## Figures and Tables

**Table 1:** Monthly mean maximum temperature (T_max_), minimum temperature (T_min_) and total rainfall during the growing seasons of sesame in Thermi (Greece, 2016 and 2017).

Month	2016			2017		
	T_min_ (°C)	T_max_ (°C)	Rainfall (mm)	T_min_ (°C)	T_max_ (°C)	Rainfall (mm)
May	9	32	75.2	11	30	5.8
June	14	38	13.3	14	39	13.2
July	17	36	0.7	16	40	78.3
August	18	36	56.6	14	38	12.9
September	9	31	80	12	35	2.7
Mean	13.4	34.6		13.4	36.4	-
Total	-	-	225.8	-	-	158.9

**Table 2:** Eigenvalues for the first eight most significant components (F1–F8) of the principal component analysis (PCA) for 37 sesame cultivars.

	F1	F2	F3	F4	F5	F6	F7	F8
**Eigenvalue**	7.13	4.49	3.87	1.82	1.39	1.19	1.14	1.04
**Variability (%)**	25.48	16.03	13.83	6.48	4.98	4.26	4.08	3.71
**Cumulative (%)**	25.48	41.51	55.34	61.82	66.8	71.06	75.14	78.85

**Table 3:** Diversity statistics for seven expressed sequence tag-simple sequence repeat (EST-SSR) loci studied in 35 sesame landraces. Data are presented as mean values ± standard errors for each locus.

Locus	I	h	uh	PIC	HRM Profiles
ZM_2	0.315 ± 0.053	0.181 ± 0.039	0.186 ± 0.041	0.87	7
ZM_10	0.305 ± 0.09	0.186 ± 0.069	0.192 ± 0.072	0.65	10
ZM_11	0.276 ± 0.046	0.154 ± 0.033	0.158 ± 0.034	0.85	11
ZM_21	0.29 ± 0.052	0.168 ± 0.037	0.17 ± 0.038	0.84	8
ZM_22	0.345 ± 0.072	0.21 ± 0.06	0.21 ± 0.06	0.78	8
ZM_34	0.339 ± 0.05	0.197 ± 0.038	0.203 ± 0.039	0.85	7
ZM_47	0.268 ± 0.045	0.148 ± 0.032	0.153 ± 0.033	0.89	11

I = Shannon’s information index = –1 × (p * Ln (p) + q × Ln(q)), h = Diversity = 1 – (p^2^ + q^2^), uh = Unbiased diversity = (N/(N − 1)) × h, PIC= Polymorphism information content = 1 − Σ(p)^2^, HRM= High resolution melting analysis.

**Table 4:** The 37 sesame landraces used in this work and their geographic origin.

Landraces	Country of Origin	Landraces	Country of Origin
LIMNOS SILVER	Greece (GR)	SESA 2	Iraq (IRQ)
LIMNOS BLUE	Greece (GR)	SESA 5	Iraq (IRQ)
LIMNOS RED	Greece (GR)	SESA 9	Iraq (IRQ)
LIMNOS BLACK	Greece (GR)	SESA 4	Korea (PRK)
LIMNOS	Greece (GR)	SESA 6	Korea (PRK)
EVROS-1	Greece (GR)	SESA 7	Tajikistan (TJK)
EVROS-2	Greece (GR)	SESA 8	Nepal (NPL)
KILKIS	Greece (GR)	SESA 16	Yemen (YEM)
THERMI	Greece (GR)	SESA 17	Yemen (YEM)
STRIMONIKO	Greece (GR)	SESA 18	Yemen (YEM)
NIKOKLEIA	Cyprus (CY)	SESA 19	Yemen (YEM)
SADOVO-1	Bulgaria (BG)	SESA 20	Yemen (YEM)
SOFIA	Bulgaria (BG)	SESA 21	Yemen (YEM)
NEVENA	Bulgaria (BG)	SESA 22	Yemen (YEM)
AIDA	Bulgaria (BG)	SESA 23	Yemen (YEM)
MILENA	Italy (ΙΤ)	SESA 24	Yemen (YEM)
TOURKIAS	Turkey (TR)	SESA 25	Yemen (YEM)
		SESA 26	Yemen (YEM)
		SESA 27	Yemen (YEM)
		SESA 28	Yemen (YEM)

## Data Availability

Data is contained within the article or supplementary material.

## Supplementary Materials

The following are available online at https://www.mdpi.com/article/10.3390/plants10040656/s1, Table S1: Morpho-physiological characterization of the 37 sesame landraces based on the UPOV 2013 guidelines for the contact of tests for distinctness, uniformity, and stability of *Sesamum indicum* L. States of expression are given for defining and harmonizing the description of each qualitative characteristic for the different landraces. Each state of expression is allocated a corresponding numerical value. Table S2: Spearman’s correlation matrix of 26 morpho-physiological traits for the 37 sesame cultivars.

## References

[1] FAOSTAT Production—Crops. http://www.fao.org/faostat/en/#compare.

[2] Prasad M.N.N., Sanjay K.R., Prasad S.D. (2012). A Review on Nutritional and Nutraceutical Properties of Sesame. J. Nutr. Food Sci..

[3] Costa F.T., Neto S.M., Bloch C., Franco O.L. (2007). Susceptibility of Human Pathogenic Bacteria to Antimicrobial Peptides from Sesame Kernels. Curr. Microbiol..

[4] Anilakumar K.R., Pal A., Khanum F., Bawa A.S. (2010). Nutritional, medicinal and industrial uses of sesame (*Sesamum indicum L*.) seeds—An overview. Agric. Conspec. Sci..

[5] Elleuch M., Besbes S., Roiseux O., Blecker C., Attia H. (2007). Quality characteristics of sesame seeds and by-products. Food Chem..

[6] Wei X., Zhu X., Yu J., Wang L., Zhang Y., Li D., Zhou R., Zhang X. (2016). Identification of Sesame Genomic Variations from Genome Comparison of Landrace and Variety. Front. Plant. Sci..

[7] Dossa K., Diouf D., Wang L., Wei X., Zhang Y., Niang M., Fonceka D., Yu J., Mmadi M.A., Yehouessi L.W. (2017). The emerging oilseed crop *Sesamum indicum* enters the “Omics” era. Front. Plant Sci..

[8] Dossa K., Mmadi M.A., Zhou R., Zhang T., Su R., Zhang Y., Wang L., You J., Zhang X. (2019). Depicting the Core Transcriptome Modulating Multiple Abiotic Stresses Responses in Sesame (*Sesamum indicum* L.). Int. J. Mol. Sci..

[9] Ganopoulos I., Mylona P., Mellidou I., Kalivas A., Bosmali I., Kontzidou S., Osathanunkul M., Madesis P. (2018). Microsatellite genotyping and molecular screening of pea (*Pisum sativum* L.) germplasm with high-resolution melting analysis for resistance to powdery mildew. Plant. Gene.

[10] Furat S., Uzun B. (2010). The use of agro-morphological characters for the assessment of genetic diversity in sesame (*Sesamum indicum* L.). Plant Omics.

[11] Zhang Y., Zhang X., Che Z., Wang L., Wei W., Li D. (2012). Genetic diversity assessment of sesame core collection in China by phenotype and molecular markers and extraction of a mini-core collection. BMC Genet..

[12] Wei X., Liu K., Zhang Y., Feng Q., Wang L., Zhao Y., Li D., Zhao Q., Zhu X., Zhu X. (2015). Genetic discovery for oil production and quality in sesame. Nat. Commun..

[13] Iqbal A. (2016). Genetic estimates and diversity study in Sesame (*Sesamum indicum* L.). IOSR J. Agric. Veter. Sci..

[14] Pérez P.F., López J.F. (2009). Morphological and phenological description of 38 sweet chestnut cultivars (*Castanea sativa* Miller) in a contemporary collection. Span. J. Agric. Res..

[15] De Oliveira E.J., Dias N.L.P., Dantas J.L.L. (2011). Selection of morpho-agronomic descriptors for characterization of papaya cultivars. Euphytica.

[16] Mehmood A., Jaskani M.J., Khan I.A., Ahmad S., Ahmad R., Luo S., Ahmad N.M. (2014). Genetic diversity of Pakistani guava (*Psidium guajava* L.) germplasm and its implications for conservation and breeding. Sci. Hortic..

[17] Ganopoulos I., Tourvas N., Xanthopoulou A., Aravanopoulos F.A., Avramidou E., Zambounis A., Tsaftaris A., Madesis P., Sotiropoulos T., Koutinas N. (2018). Phenotypic and molecular characterization of apple (Malus × domestica Borkh) genetic resources in Greece. Sci. Agricola.

[18] Ramprasad E., Senthilvel S., Jatoth J.L., Yamini K.N., Dangi K.S., Ranganatha A.R.G., Varaprasad K.S. (2017). An insight into morphological and molecular diversity in Indian sesame cultivars. Indian J. Genet. Plant. Breed..

[19] Bhattacharjee M., Iqbal A., Singha S., Nath D., Prakash S., Dasgupta T. (2019). Genetic diversity in *Sesamum indicum* L.. Bangladesh J. Bot..

[20] Pandey P., Ramegowda V., Senthil-Kumar M. (2015). Shared and unique responses of plants to multiple individual stresses and stress combinations: Physiological and molecular mechanisms. Front. Plant. Sci..

[21] Bhat K.V., Babrekar P.P., Lakhanpaul S. (1999). Study of genetic diversity in Indian and exotic sesame (*Sesamum indicum* L.) germplasm using random amplified polymorphic DNA (RAPD) markers. Euphytica.

[22] Ercan A.G., Taskin K.M., Turgut K. (2004). Analysis of genetic diversity in Turkish sesame (*Sesamum indicum* L.) populations using RAPD markers. Genet. Resour. Crop. Evol..

[23] Arriel N.H.C., Di Mauro A.O., Arriel E.F., Unêda-Trevisoli S.H., Costa M.M., Bárbaro I.M., Muniz F.R.S. (2007). Genetic divergence in sesame based on morphological and agronomic traits. Crop. Breed. Appl. Biotechnol..

[24] Salazar B., Laurentín H., Dávila M., Castillo M.A. (2006). Reliability of the rapd technique for germplasm analysis of sesame (*Sesamum indicum* L) from Venezuela. Interciencia.

[25] Abdellatef E., Sirelkhatem R., Mohamed Ahmed M.M., Radwan K.H., Khalafalla M.M. (2008). Study of genetic diversity in Sudanese sesame (*Sesamum indicum* L.) germplasm using random amplified polymorphic DNA (RAPD) markers. Afr. J. Biotechnol..

[26] Uzun B., Lee D., Donini P., Çaǧirgan M.L. (2003). Identification of a molecular marker linked to the closed capsule mutant trait in sesame using AFLP. Plant. Breed..

[27] Laurentin E.H., Karlovsky P. (2006). Genetic relationship and diversity in a sesame (*Sesamum indicum* L.) germplasm collection using amplified fragment length polymorphism (AFLP). BMC Genet..

[28] Laurentin H., Karlovsky P. (2007). AFLP fingerprinting of sesame (*Sesamum indicum* L.) cultivars: Identification, genetic relationship and comparison of AFLP informativeness parameters. Genet. Resour. Crop. Evol..

[29] Kim D.H., Zur G., Danin-Poleg Y., Lee S.W., Shim K.B., Kang C.W., Kashi Y. (2002). Genetic relationships of sesame germplasm collection as revealed by inter-simple sequence repeats. Plant. Breed..

[30] Parsaeian M., Mirlohi A., Saeidi G. (2011). Study of genetic variation in sesame (*Sesamum indicum* L.) using agro-morphological traits and ISSR markers. Russ. J. Genet..

[31] Dixit A., Jin M.-H., Chung J.-W., Yu J.-W., Chung H.-K., Ma K.-H., Park Y.-J., Cho E.-G. (2005). Development of polymorphic microsatellite markers in sesame (*Sesamum indicum* L.). Mol. Ecol. Notes.

[32] Sudhakara V., Kola R., Yepuri V., Surapaneni M. (2012). Genetic Diversity and DNA Fingerprinting in Sesame (*Sesamum indicum* L.). Cultiv. Angrau..

[33] Uncu A. (2015). Özgür; Gultekin, V.; Allmer, J.; Frary, A.; Doganlar, S. Genomic Simple Sequence Repeat Markers Reveal Patterns of Genetic Relatedness and Diversity in Sesame. Plant. Genome.

[34] Dossa K., Wei X., Zhang Y., Fonceka D., Yang W., Diouf D., Liao B., Cisse N., Zhang X. (2016). Analysis of Genetic Diversity and Population Structure of Sesame Accessions from Africa and Asia as Major Centers of Its Cultivation. Genes.

[35] Sehr E.M., Okello-Anyanga W., Hasel-Hohl K., Burg A., Gaubitzer S., Rubaihayo P.R., Okori P., Vollmann J., Gibson P., Fluch S. (2016). Assessment of genetic diversity amongst Ugandan sesame (*Sesamum indicum* L.) landraces based on agromorphological traits and genetic markers. J. Crop. Sci. Biotechnol..

[36] Suh M.C., Kim M.J., Hur C.-G., Bae J.M., Park Y.I., Chung C.-H., Kang C.-W., Ohlrogge J.B. (2003). Comparative analysis of expressed sequence tags from *Sesamum indicum* and Arabidopsis thaliana developing seeds. Plant. Mol. Biol..

[37] Wei L.-B., Zhang H.-Y., Zheng Y.-Z., Guo W.-Z., Zhang T.-Z. (2008). Developing EST-Derived Microsatellites in Sesame (*Sesamum indicum* L.). Acta Agron. Sin..

[38] Wei W., Qi X., Wang L., Zhang Y., Hua W., Li D., Lv H., Zhang X. (2011). Characterization of the sesame (*Sesamum indicum* L.) global transcriptome using Illumina paired-end sequencing and development of EST-SSR markers. BMC Genom..

[39] Ke T., Dong C., Mao H., Zhao Y., Chen H., Liu H., Dong X., Tong C., Liu S. (2011). Analysis of expression sequence tags from a full-length-enriched cDNA library of developing sesame seeds (*Sesamum indicum*). BMC Plant. Biol..

[40] Wu K., Yang M., Liu H., Tao Y., Mei J., Zhao Y. (2014). Genetic analysis and molecular characterization of Chinese sesame (Sesamum indicum L.) cultivars using Insertion-Deletion (InDel) and Simple Sequence Repeat (SSR) markers. BMC Genet..

[41] Badri J., Yepuri V., Ghanta A., Siva S., Siddiq E.A. (2014). Development of microsatellite markers in sesame (*Sesamum indicum* L.). Turk. J. Agric. For..

[42] Nandakumar N., Singh A., Sharma R., Mohapatra T., Prabhu K., Zaman F. (2004). Molecular fingerprinting of hybrids and assessment of genetic purity of hybrid seeds in rice using microsatellite markers. Euphytica.

[43] Kaur G., Joshi A., Jain D. (2018). SSR-Marker assisted evaluation of Genetic Diversity in Mungbean (Vigna radiata (L.) Wilcezk) genotypes. Braz. Arch. Biol. Technol..

[44] Zhang Y.-X., Zhang X.-R., Hua W., Wang L.-H., Che Z. (2010). Analysis of genetic diversity among indigenous landraces from sesame (*Sesamum indicum* L.) core collection in China as revealed by SRAP and SSR markers. Genes Genom..

[45] Thomson M.J., Septiningsih E.M., Suwardjo F., Santoso T.J., Silitonga T.S., McCouch S.R. (2007). Genetic diversity analysis of traditional and improved Indonesian rice (Oryza sativa L.) germplasm using microsatellite markers. Theor. Appl. Genet..

[46] Bhat K., Kumari R., Pathak N., Rai A. (2014). Value addition in sesame: A perspective on bioactive components for enhancing utility and profitability. Pharmacogn. Rev..

[47] (2013). UPOV International Union for the Protection of New Varieties of Plants (Sesame). https://www.upov.int/portal/index.html.en.

[48] Kaiser H.F. (1960). The Application of Electronic Computers to Factor Analysis. Educ. Psychol. Meas..

[49] Pandey S.K., Das A., Rai P., Dasgupta T. (2015). Morphological and genetic diversity assessment of sesame (*Sesamum indicum* L.) accessions differing in origin. Physiol. Mol. Biol. Plants.

[50] Prasad R., Gangopadhyay G. (2011). Phenomic analyses of Indian and exotic accessions of Sesame *(Sesamum indicum* L.). J. Plant Breed. Crop Sci..

[51] Jeyaraj J., Beevy S.S. (2020). A Comparative Study on the Reproductive Success of Two Species of Sesamum L. (Pedaliaceae). Adv. Zool. Bot..

[52] Tabatabaei I., Pazouki L., Bihamta M.R., Mansoori S., Javaran M.J., Niinemets Ü. (2011). Genetic variation among iranian sesame (*Sesamum indicum* L.) accessions vis-à-vis exotic genotypes on the basis of morpho-physiological traits and RAPD markers. Aust. J. Crop Sci..

[53] Tahmasebi A., Ashrafi-Dehkordi E., Shahriari A.G., Mazloomi S.M., Ebrahimie E. (2019). Integrative meta-analysis of transcriptomic responses to abiotic stress in cotton. Prog. Biophys. Mol. Biol..

[54] Cho Y.-I., Park J.-H., Lee C.-W., Ra W.-H., Chung J.-W., Lee J.-R., Ma K.-H., Lee S.-Y., Lee K.-S., Lee M.-C. (2011). Evaluation of the genetic diversity and population structure of sesame (*Sesamum indicum* L.) using microsatellite markers. Genes Genom..

[55] Dossa K., Li D., Wang L., Zheng X., Liu A., Yu J., Wei X., Zhou R., Foncéka D., Diouf D. (2017). Transcriptomic, biochemical and physio-anatomical investigations shed more light on responses to drought stress in two contrasting sesame genotypes. Sci. Rep..

[56] Olusola T.O., Opeyemi A.O., Oluwabukola O.A., Abiodun S., Omowumi A.A., Oyejide S.O., Oduoye O.T., Oluwasanya O.A., Arikawe O.O., Sunday A. (2020). Genetic variation via SSR polymorphic information content and ecological distribution of Nigerian sesame. Afr. J. Biotechnol..

[57] Woldesenbet D.T., Kassahun T., Endashaw B. (2015). Genetic diversity of sesame germplasm collection (*Sesamum indicum* L.): Implication for conservation, improvement and use. Int. J. Biotechnol. Mol. Biol. Res..

[58] Pham T.D., Geleta M., Bui T.M., Bui T.C., Merker A., Carlsson A.S. (2011). Comparative analysis of genetic diversity of sesame (*Sesamum indicum* L.) from Vietnam and Cambodia using agro-morphological and molecular markers. Hereditas.

[59] Metsalu T., Vilo J. (2015). ClustVis: A web tool for visualizing clustering of multivariate data using Principal Component Analysis and heatmap. Nucleic Acids Res..

[60] Doyle J.J., Doyle J.L. (1987). A rapid DNA isolation procedure for small quantities of fresh leaf tissue. Phytochemistry.

[61] Peakall R., Smouse P.E. (2012). GenAlEx 6.5: Genetic analysis in Excel. Population genetic software for teaching and research - an update. Bioinformatics.

[62] Kumar S., Stecher G., Li M., Knyaz C., Tamura K. (2018). MEGA X: Molecular evolutionary genetics analysis across computing platforms. Mol. Biol. Evol..

